# Speech-based concussion detection in athletes using Mel-spectrograms and convolutional neural networks

**DOI:** 10.3389/fneur.2026.1830075

**Published:** 2026-07-16

**Authors:** Rahmina Rubaiat, Christian Poellabauer

**Affiliations:** Mobile Sensing and Analytics Lab, Knight Foundation School of Computing and Information Sciences, Florida International University, Miami, FL, United States

**Keywords:** concussion detection, convolutional neural networks, deep learning, Grad-CAM, neurological disorders, speech biomarkers

## Abstract

Neurological disorders can alter motor control, cognition, and speech production in subtle ways that are difficult to detect using standard diagnostic tools. Because conventional imaging techniques such as CT and MRI often fail to reveal functional abnormalities, there is growing interest in non-invasive digital health approaches. This study investigates the potential of speech-based deep learning for detecting concussion-related dysfunction using structured speech tasks. Audio recordings from 225 high school and college athletes (approximately 200 retained after quality filtering) performing eight speech tasks were analyzed. The recordings were converted into Mel spectrograms and processed using a convolutional neural network (CNN), with data augmentation applied to improve model generalization. The results demonstrate strong classification performance, particularly for tasks requiring dynamic articulation and rhythmic control. To better interpret model behavior, the speech tasks were grouped into three functional categories and examined using gradient-weighted class activation mapping (Grad-CAM). Sentence and word reading tasks, as well as rapid syllable repetition, achieved the highest accuracy (up to 96%) and exhibited focused activation patterns associated with phonetic complexity and motor timing. In contrast, sustained vowel tasks produced lower performance and more diffuse model attention, likely due to their limited acoustic variability. These findings highlight the effectiveness of CNN-based analysis of dynamic speech features and emphasize the importance of task selection in speech-based digital diagnostics. Although the framework may inform future speech-based neurological assessment research, this study focuses on mild traumatic brain injury (concussion) as an initial use case.

## 1 Introduction

Concussion, a form of mild traumatic brain injury (mTBI), can disrupt brain function in subtle and heterogeneous ways that are not always visible on routine structural imaging such as CT or MRI (1, 2). In sports-related settings, timely identification of concussion is important because delayed detection may prolong symptoms and increase the risk of adverse cognitive, behavioral, and motor outcomes (1). However, concussion assessment often relies on symptom reports, clinical examination, and related screening tools, which may vary across individuals and may not fully capture subtle functional abnormalities (1, 3). These challenges have motivated growing interest in non-invasive digital biomarkers that can support more objective and scalable concussion assessment.

Speech is a particularly promising candidate for such assessment because it reflects complex interactions among cognitive, motor, linguistic, and sensory processes regulated by the brain. Even mild neurological disruption may affect articulation, speech timing, coordination, prosody, and fluency in measurable ways (4–8). In the context of concussion, prior studies have reported changes in speech rate, articulatory precision, and coordination of the speech musculature, suggesting that structured speech tasks may reveal subtle concussion-related dysfunction (9–14). Because these alterations may emerge even when routine imaging is unrevealing, speech-based analysis offers a promising non-invasive complement to conventional clinical evaluation.

### 1.1 Speech as a biomarker for concussion

Human speech requires tightly coordinated control of respiration, phonation, articulation, timing, and cognitive-linguistic planning. As a result, neurological disorders often produce measurable changes in speech production. Research in conditions such as Alzheimer's disease, Parkinson's disease, and multiple sclerosis has shown that alterations in articulation, fluency, and vocal stability can reflect underlying neural dysfunction (4–8). These findings support the broader use of speech analysis as a digital biomarker in neurological health research.

For concussion specifically, the expected speech changes are especially relevant to motor-speech control. Concussion may affect neural systems involved in motor planning, temporal coordination, articulatory sequencing, and cognitive-linguistic control, which in turn may influence speech production during structured tasks (9, 10, 12). Tasks that require rapid transitions between speech sounds, accurate timing, prosodic modulation, or coordinated oral movements may therefore be more sensitive to concussion-related disruption than more stationary phonatory tasks. This perspective provides a neurobiologically grounded rationale for comparing multiple speech tasks rather than relying on a single vowel-based assessment.

### 1.2 Deep learning for speech analysis

Advances in machine learning have substantially improved the analysis of complex biomedical signals, including speech. Earlier approaches to speech-based health assessment often relied on conventional machine learning models such as support vector machines trained on handcrafted acoustic features. Although useful, these approaches depend heavily on feature engineering and domain expertise. Deep learning models, particularly convolutional neural networks (CNNs), offer an alternative by learning hierarchical representations directly from time-frequency inputs such as spectrograms (15–17).

CNN-based models have shown strong performance in medical speech analysis, including pathological voice detection, Parkinsonian speech analysis, and clinical audio screening (18–20). In concussion research, this is particularly appealing because the relevant speech changes may be subtle, distributed, and task-dependent rather than easily captured by a small predefined feature set. In addition, explainable AI techniques such as gradient-weighted class activation mapping (Grad-CAM) can improve interpretability by highlighting the spectro-temporal regions that contribute most strongly to model decisions (21).

### 1.3 Speech representations for neural models

The choice of speech representation is critical for deep learning-based audio analysis. Mel spectrograms are widely used because they capture the distribution of acoustic energy across perceptually motivated frequency bands over time, while preserving rich spectral and temporal structure. Compared with handcrafted descriptors such as formants, pitch contours, jitter, or shimmer, mel spectrograms allow neural models to learn discriminative acoustic patterns directly from data in a scalable and data-driven manner (16, 17).

Mel-spectrogram-based CNN architectures have been successfully applied in a range of medical speech tasks, including Parkinson's disease detection, cognitive impairment screening, and pathological voice analysis (5, 18, 20, 22, 23). These findings suggest that mel spectrograms are well suited for identifying subtle speech changes associated with concussion in structured assessment tasks.

### 1.4 Data augmentation

One of the main challenges in deep learning for clinical speech analysis is the limited availability of labeled data. This issue is especially relevant in concussion research, where collecting clinically validated recordings can be time-consuming and resource-intensive. Small datasets increase the risk of overfitting, reducing generalizability to unseen recordings. Data augmentation has therefore become an important strategy for improving model robustness in speech analysis.

Common augmentation approaches include adding Gaussian noise, modifying speaking rate, and applying spectrogram-level perturbations to simulate realistic recording variability (16, 17, 24). These techniques have improved performance in a range of medical audio tasks, including pathological voice detection, respiratory sound classification, and neurological speech analysis (20, 25). In concussion datasets, augmentation is particularly useful because clinically labeled recordings are limited and between-subject variability can be substantial.

### 1.5 Speech task design for concussion assessment

The design of the speech task is a critical factor in speech-based concussion assessment because different tasks place different demands on articulation, motor coordination, timing, respiration, and cognitive-linguistic planning. Early studies often relied on limited task types, such as sustained vowel phonation, which primarily capture phonatory stability but may overlook broader motor-speech or cognitive deficits (13). However, concussion-related dysfunction may affect multiple dimensions of speech production, including articulatory timing, coordination, prosody, and respiratory control (12, 14).

Motor-speech tasks such as diadochokinetic (DDK) syllable repetition are especially relevant because they probe rapid articulatory sequencing and speech motor coordination, both of which may be affected following brain injury (26, 27). Likewise, structured reading tasks may capture deficits in prosodic modulation, phonetic transitions, and cognitive-linguistic planning (28, 29). By contrast, sustained vowel tasks mainly reflect vocal steadiness and respiratory-phonatory control within a relatively stationary acoustic pattern.

These task differences are important not only clinically but also computationally. Tasks involving richer temporal-spectral variation, rapid articulatory transitions, and stronger coordination demands may provide more discriminative information for CNN-based classification than sustained phonation tasks. We therefore hypothesize that dynamic speech tasks, such as reading and DDK repetition, will be more informative than sustained vowels for speech-based concussion detection.

### 1.6 Study objective and contributions

In this study, we investigate whether structured speech recordings can be used to distinguish athletes with clinically confirmed concussion from healthy control or baseline recordings using mel-spectrogram representations and a convolutional neural network (CNN). The analysis is based on eight structured speech tasks that differ in articulatory, prosodic, and motor-speech demands.

The contributions of this work are three-fold. First, we evaluate the effectiveness of a mel-spectrogram-based CNN for speech-based concussion detection across multiple structured task types. Second, we compare the relative discriminative value of articulation-rich reading tasks, DDK motor-speech tasks, and sustained vowel phonation tasks. Third, we use Grad-CAM visualizations to examine which spectro-temporal regions contribute most strongly to model predictions across these task groups.

Rather than presenting a general neurological screening framework, this study should be interpreted as a proof-of-concept investigation of speech-based concussion detection in a young athlete cohort. By focusing on task-specific performance and model interpretability, this work aims to inform the development of more targeted, clinically grounded, and non-invasive speech-based tools for concussion assessment.

## 2 Material and methods

In this study, we employed a convolutional neural network (CNN) to classify speech recordings from concussed and healthy control participants. Speech signals were first converted into mel spectrogram representations and then used as inputs to a CNN-based classification model. The overall workflow consisted of data collection, preprocessing, mel spectrogram generation, data augmentation, model training, and cross-validation-based evaluation.

### 2.1 Neurological speech tasks

Each participant completed eight structured neurological speech tasks commonly used in concussion and motor-speech assessments. These tasks are traditionally evaluated using repetition counts, timing, or perceptual scoring by clinicians. In this study, however, we focused on analyzing the acoustic and articulatory characteristics of the recordings using spectrogram-based representations.

The tasks and their behavioral descriptions are summarized in Table 1. These tasks capture different aspects of speech production, including articulation, fluency, phonatory control, and motor coordination. For example, dynamic articulation tasks such as word and sentence reading require natural speech production and prosody. Diadochokinetic (DDK) tasks require participants to repeat syllables as quickly and accurately as possible, typically for 5–12 s. Sustained vowel tasks require participants to hold a vowel sound for as long and as steadily as possible, capturing information related to vocal stability and respiratory control.

**Table 1:** Grouping of neurological speech tests with descriptions and rationale.

Test	Speaking assignment	Detailed description and rationale	Group
1	Participate, application, education, difficulty, congratulation, possibility, mathematical, opportunity	Word reading test: participant reads the printed words aloud at a natural pace; captures articulatory precision, prosody, and fluency and detects subtle speech impairments caused by cognitive or neurological changes in post-concussion (28).	1
2	Put the book here	Stress test: this task was elicited in three forms by emphasizing each target word (“PUT,” “BOOK,” “HERE”) in separate recordings to capture stress-related articulatory variation. It evaluates speech fluency, articulation, and prosody, which can reveal cognitive or motor impairments linked to concussions (29).	
3	We saw several wild animals	Sentence reading test: participant reads a short printed sentence aloud at a natural pace; captures articulatory precision, prosody, and fluency, which is important in identifying subtle neurological disruptions in post-concussion (29).	
4	Pa	Single-syllable DDK task: participant repeats “pa” as quickly and clearly as possible in one breadth; reflects lip closure and bilabial articulation speed. Also, measures neuromotor function and speech motor control by repeating single syllables rapidly, revealing motor coordination impairments due to concussion (26).	2
5	Ka	Single-syllable DDK task: participant repeats “pa” as quickly and clearly as possible in one breadth; reflects posterior tongue motion and velar articulation. Similar to Test 4, assesses speech motor control through single syllable repetitions, indicating potential neuromotor disruptions post-concussion (26).	
6	PaTaKa	Alternating DDK task: participant repeats “pa-ta-ka” as fast and as accurately as possible in one breadth duration, evaluates motor speech coordination and agility, sensitive to detecting motor speech disorders caused by concussions (27). Traditionally scored by counting repetitions/second, here it was analyzed acoustically via spectrogram patterns.	
7	Sustained vowel (Aa)	Sustained vowel test: participant sustains the vowel /a/ for as long and as steadily as possible in one breadth, revealing phonatory stability and respiratory control. Assesses voice quality, steadiness, and vocal tremor, useful for detecting subtle concussion-related speech changes after concussion (30).	3
8	Sustained vowel (Uu)	Sustained vowel test: similar to Test 7, participant sustains the vowel /u/ for as long and as steadily as possible in one breadth. It is sensitive to vocal tract configuration and breath support and evaluates vocal tremor and voice stability, indicative of neurological damage post-concussion (30).	

Each of these tasks evaluates specific speech characteristics associated with neurological function, including vowel articulation, diadochokinetic rate, speech fluency, and motor control (26–29). For instance, the PaTaKa task (Test 6 in Table 1) assesses motor-speech coordination, while the sustained vowel tasks (Tests 7 and 8) capture vocal tremor and voice quality, both of which may be altered following concussion. For analysis purposes, the tasks listed in Table 1 were grouped into three functional categories based on similarities in speech characteristics and motor demands.

### 2.2 Data collection

The dataset used in this study was collected by researchers at the University of Notre Dame during the 2014–2015 sports seasons. More than 2,500 youth athletes at the high school and collegiate levels completed the eight neurological speech tasks using a custom tablet-based recording application. Among these participants, 123 athletes (106 male and 17 female) were clinically diagnosed with concussion.

The binary classification task in this study was defined as *concussion vs. control*. Positive-class recordings were obtained from athletes with clinically confirmed concussion, whereas negative-class recordings were obtained from baseline recordings collected from healthy athletes prior to the sports season. Concussion diagnoses were confirmed through standardized clinical evaluation and physician assessment in accordance with institutional sports medicine protocols.

All athletes completed baseline assessments before the start of the sports season. Follow-up recordings were obtained for selected participants, and additional recordings were collected when a concussion was suspected. For concussed participants, contextual metadata included the time of impact and the time of speech testing, recorded as UNIX timestamps. The elapsed time between these two events was computed in minutes, providing temporal context for the post-injury recordings. In the concussed cohort, speech recordings were therefore obtained after the documented time of impact, and the analyzed post-injury samples reflect speech collected within the acute-to-subacute period following concussion rather than long-term recovery. For the present analysis, only recordings corresponding to the concussion and control classes described above were used.

Speech recordings were collected using a custom mobile application that guided participants sequentially through the eight neurological tasks. Each task was recorded separately and stored in uncompressed WAV format. Recordings were captured using iPad devices equipped with external directional microphones to promote consistency in acquisition conditions across participants. The original recordings were sampled at 44.1 kHz and were resampled to 16 kHz during preprocessing to reduce computational requirements while preserving the speech information relevant to the analysis.

Following an initial quality assessment, recordings with excessive noise or recording artifacts were excluded from analysis. After quality filtering, the number of usable recordings varied slightly across speech tasks because some recordings were excluded for quality-related reasons. Consequently, the final per-test analytic sample included approximately 100 concussion recordings and approximately 100 control recordings per task.

### 2.3 Dataset summary

A summary of the dataset composition is provided in Table 2. Each participant contributed recordings for the eight structured speech tasks, and each task was treated as a separate classification setting. To ensure a consistent input size for the neural network, recordings were standardized to a duration of 12 s prior to model training.

**Table 2:** Summary of dataset composition.

Category	Participants	Total recordings	Average duration (s)	Train samples	Test samples	Description
Concussed	~100	~800	12	640	160	Recordings from athletes with clinically confirmed concussion
Healthy controls	~100	~800	12	640	160	Baseline recordings from same athletes prior to season
Total	~200	~1,600	12	1,280	320	Balanced dataset used for training and evaluation across eight tasks

To increase sample diversity and improve model robustness, Gaussian-noise-based augmentation was applied after preprocessing. After augmentation, each speech task contained approximately 550 samples per class. Model evaluation was performed using participant-level cross-validation so that recordings from the same individual were not present in both training and testing folds.

### 2.4 Data preprocessing

Prior to model training, all audio recordings underwent a standardized preprocessing pipeline. Each recording was first resampled to 16 kHz and normalized to a fixed duration of 12 s. Shorter recordings were zero-padded, whereas longer recordings were truncated, yielding a uniform input length across all samples.

To address the limited size of the dataset and improve model robustness, Gaussian noise augmentation was applied to each preprocessed recording. Following commonly used augmentation strategies in speech analysis research (11, 13, 16), zero-mean Gaussian noise with a noise factor of 0.005 was added to each recording. Each original recording was augmented five times, thereby increasing the diversity of the dataset used for model development.

**Figure 1:**
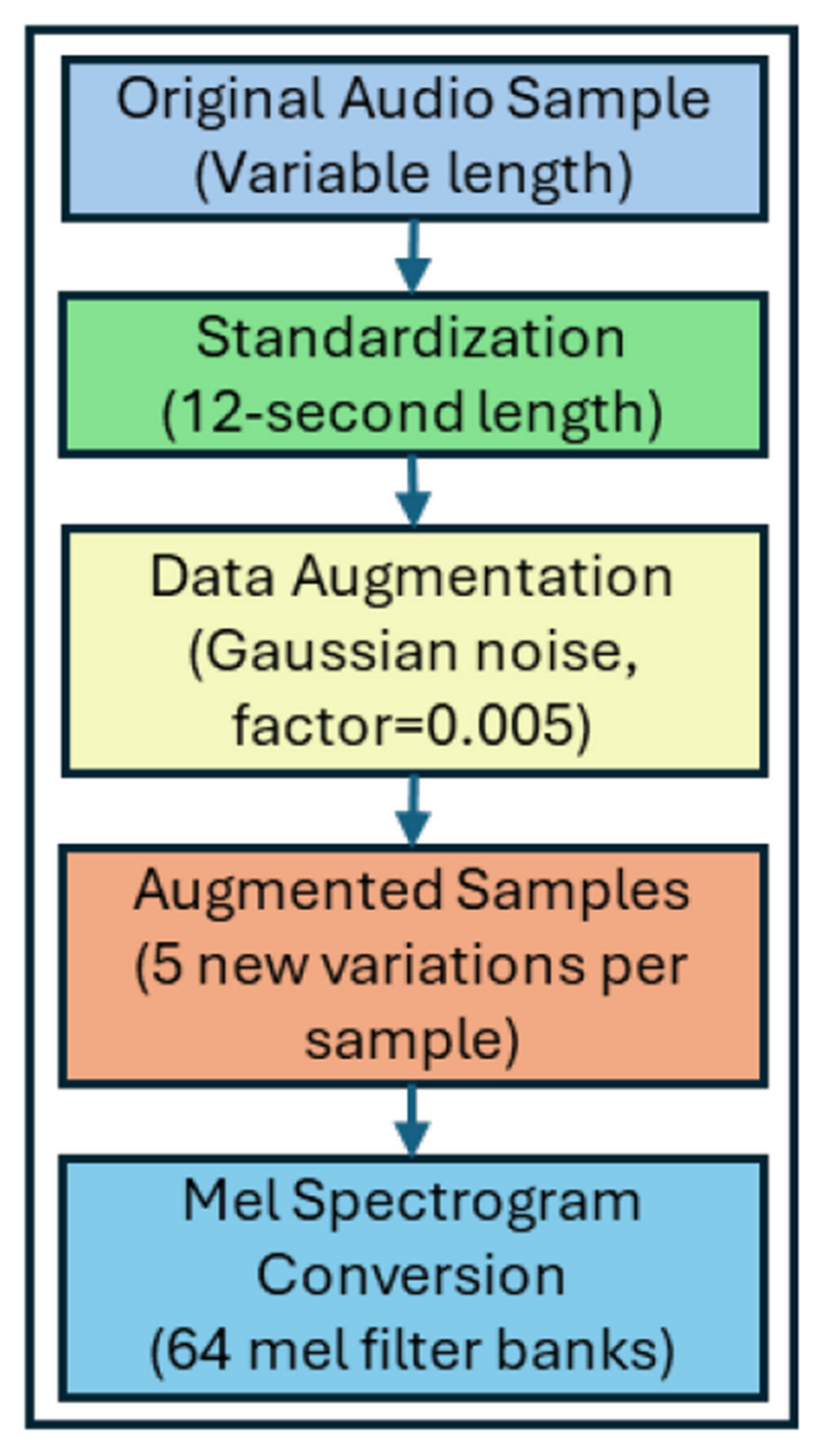
Visual summary of the preprocessing pipeline for speech analysis.

After preprocessing and augmentation, each audio recording was transformed into a mel spectrogram using 64 mel filter banks. This time–frequency representation captures the distribution of acoustic energy over time and frequency and enables the CNN to learn discriminative patterns associated with concussion-related speech changes. The overall preprocessing pipeline, including duration standardization, noise augmentation, and mel spectrogram generation, is illustrated in Figure 1.

### 2.5 Model architecture

The CNN architecture used in this study processes mel spectrogram inputs using a series of convolutional layers designed to capture hierarchical time–frequency features. CNNs are well suited for spectrogram-based analysis because they can learn localized acoustic patterns that may correspond to articulatory or phonatory abnormalities associated with concussion-related speech changes (19).

The proposed model consists of three convolutional layers with filter sizes of 16, 32, and 64, respectively. Each convolutional layer uses a 3 × 3 kernel followed by batch normalization, ReLU activation, and 2 × 2 max pooling to progressively extract higher-level acoustic features while reducing spatial dimensionality. The output of the convolutional layers is flattened and passed through a fully connected dense layer with 128 neurons. Dropout with a rate of 0.5 is applied to reduce overfitting. The final output layer uses softmax activation to perform binary classification between concussion and control speech recordings.

**Figure 2:**
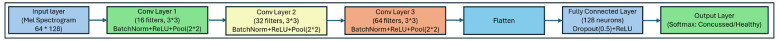
Model architecture for concussion detection using speech-based Mel spectrogram inputs.

A schematic representation of the CNN architecture is shown in Figure 2.

### 2.6 Training procedure

The CNN model was trained and evaluated using five-fold cross-validation to obtain stable performance estimates. In each fold, the model was trained on four subsets of the data and evaluated on the remaining subset. To reduce the risk of subject-level leakage, cross-validation was performed at the participant level, ensuring that recordings from the same individual appeared in only one-fold.

Model training was performed using the Adam optimizer with an initial learning rate of 0.001. A learning-rate scheduler was used to adjust the learning rate based on validation loss. Early stopping with a patience of three epochs was applied to reduce overfitting and terminate training when validation performance no longer improved.

### 2.7 Evaluation metrics

Model performance was evaluated using accuracy, precision, recall, F1 score, and confusion matrix analysis. These metrics provide complementary perspectives on classification performance, including overall correctness, positive predictive value, sensitivity to concussion cases, and the balance between precision and recall. The definitions and descriptions of these metrics are summarized in Table 3. Performance values were computed for each cross-validation fold and then averaged to obtain the overall results reported in this study.

**Table 3:** Evaluation metrics and definitions for assessing model performance in concussion detection.

Metric	Definition
Accuracy	Proportion of correctly classified samples over the total.
Precision	Proportion of true positive predictions among all positive predictions, highlighting model accuracy.
Recall	Proportion of true positive predictions among actual positive cases, reflecting sensitivity to concussed cases.
F1 score	Harmonic mean of precision and recall, balancing the model's ability to identify concussed and healthy cases.
Confusion matrix	Breakdown of true positives, true negatives, false positives, and false negatives, providing insights into model biases and classification challenges.

## 3 Results

### 3.1 Performance metrics summary

**Figure 3:**
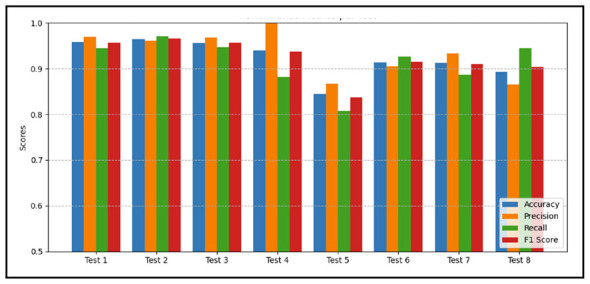
Summary of performance metrics for concussion detection across eight tests.

The quantitative performance results of the proposed CNN-based concussion detection model across the eight neurological speech tests are summarized in Table 4 and illustrated in Figure 3. The results demonstrate consistently high classification performance across most tasks, particularly in Tests 1 through 4, where accuracy exceeded 94% with balanced precision and recall values.

**Table 4:** Summary of performance metrics for concussion detection across eight tests.

Tests	Accuracy	Precision	Recall	F1 score
Test 1	0.9585	0.9699	0.9444	0.9570
Test 2	**0.9648**	0.9609	**0.9704**	**0.9657**
Test 3	0.9565	0.9680	0.9473	0.9575
Test 4	0.9401	**1.0000**	0.8818	0.9372
Test 5	0.8443	0.8674	0.8080	0.8367
Test 6	0.9134	0.9046	0.9267	0.9155
Test 7	0.9130	0.9342	0.8869	0.9099
Test 8	0.8932	0.8659	0.9449	0.9037

Performance metrics which got more than 0.95.

For example, Test 1 achieved an accuracy of 95.85% and an F1 score of 95.70%, indicating strong generalization capability and reliable concussion detection performance.

Test 2 achieved the highest recall at 97.04%, underscoring the model's effectiveness in identifying concussed participants, while precision remained stable at 96.09%. Test 4 achieved a perfect precision score of 100%, indicating no false positives, although recall was slightly lower at 88.18%, suggesting a more conservative classification behavior.

Tests 5 through 8 exhibited slightly lower performance, with Test 5 producing the lowest accuracy (84.43%) and F1 score (83.67%). However, precision and recall remained relatively high across these tasks. In particular, Tests 6, 7, and 8 maintained F1 scores exceeding 90%, demonstrating the robustness of the CNN model across diverse speech task conditions.

In Figure 3, the *y*-axis scale was adjusted to range from 0.5 to 1 instead of the default range of 0 to 1. This adjustment was applied to improve visual clarity and highlight subtle differences among the performance metrics evaluated, all of which exceeded 0.8.

In general, these results highlight the ability of the CNN model to accurately distinguish between concussed and healthy controls across multiple evaluation metrics, indicating that the model was able to distinguish concussion and control recordings effectively within this dataset.

### 3.2 Confusion matrix analysis

**Figure 4:**
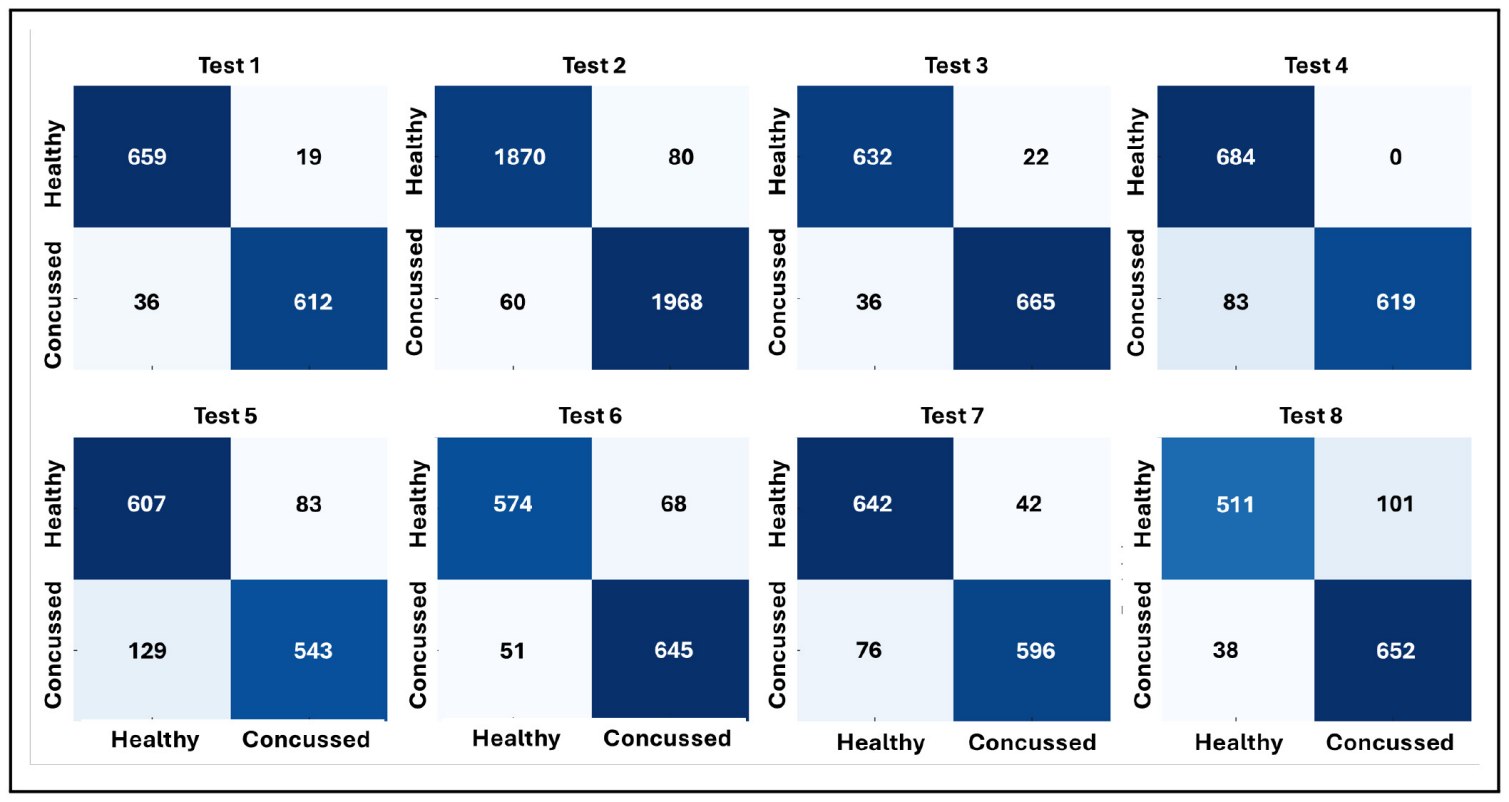
Confusion matrices for concussion detection model performance across eight tests.

To further assess the performance of the model in distinguishing between concussed and healthy individuals, confusion matrices were generated for each test, as shown in Figure 4. These matrices provide a detailed breakdown of true positives, true negatives, false positives, and false negatives across all tests, allowing deeper insight into classification patterns and potential biases.

Across the tests, the model consistently achieved high true positive and true negative counts, with relatively low numbers of false positives and false negatives. For instance, in Test 4, perfect precision (100%) was achieved without any false positives, indicating that all predicted concussion cases were correct. However, the slightly lower recall (88.18%) suggests that some concussion cases were missed, reflected by the presence of false negatives.

In contrast, Test 2 demonstrated a strong balance between precision and recall, with high true positive and true negative rates and minimal misclassification. Tests 5 through 8 exhibited slightly greater variability, particularly in Test 5 where the model produced a higher number of false negatives, corresponding to the lowest recall among the tests (80.80%). These observations highlight the importance of examining confusion matrices alongside standard evaluation metrics to better understand classification behavior.

### 3.3 Group-wise performance overview

To better understand the comparative effectiveness of different types of speech tasks for concussion detection, the eight neurological speech tests were grouped into three functional categories based on task characteristics:

**Group 1:** Sentence and word reading tasks (Tests 1–3).

**Group 2:** Diadochokinetic (DDK) motor speech tasks (Tests 4–6).

**Group 3:** Sustained vowel phonation tasks (Tests 7–8).

**Figure 5:**
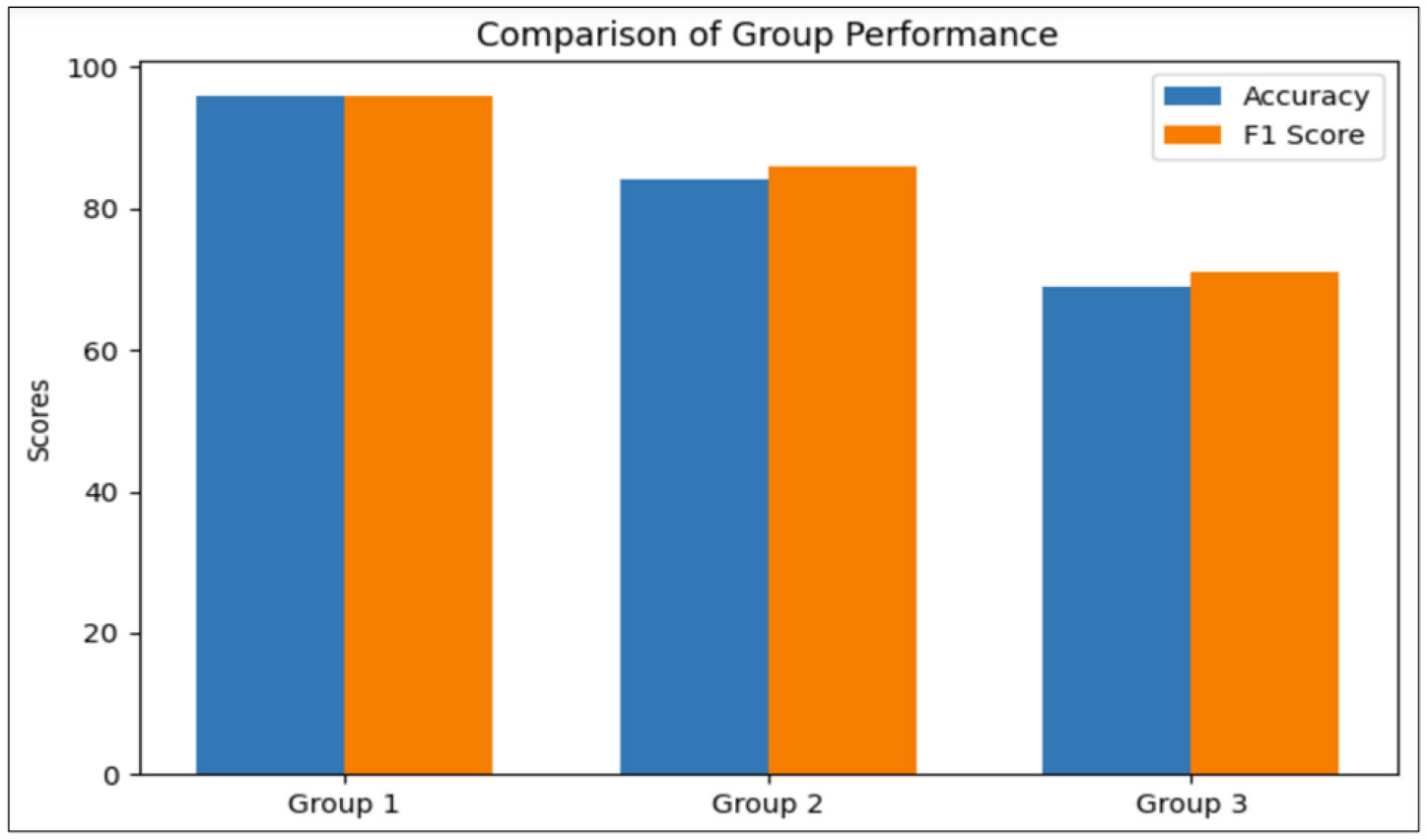
Group-wise comparison of classification accuracy and F1 score across sentence/word reading tasks (Group 1), DDK motor tasks (Group 2), and sustained vowel phonation tasks (Group 3).

Group-wise average performance metrics were computed to compare classification accuracy and F1 score across these task categories. As shown in Figure 5, Groups 1 and 2 achieved strong classification performance, with Group 1 producing the highest accuracy and F1 score. In contrast, Group 3 exhibited noticeably lower performance across both metrics.

This result suggests that speech tasks involving dynamic articulation and motor timing (Groups 1 and 2) provide more discriminative cues for CNN-based concussion detection. The reduced performance observed for sustained vowels in Group 3 motivated further investigation into their acoustic characteristics.

### 3.4 Feature analysis for group 3 tasks

The group-wise performance analysis revealed that Group 3 (sustained vowel phonation) exhibited noticeably lower classification accuracy and F1 score compared to Groups 1 and 2. To better understand this performance gap, an exploratory analysis of acoustic features in sustained vowel recordings was conducted.

**Figure 6:**
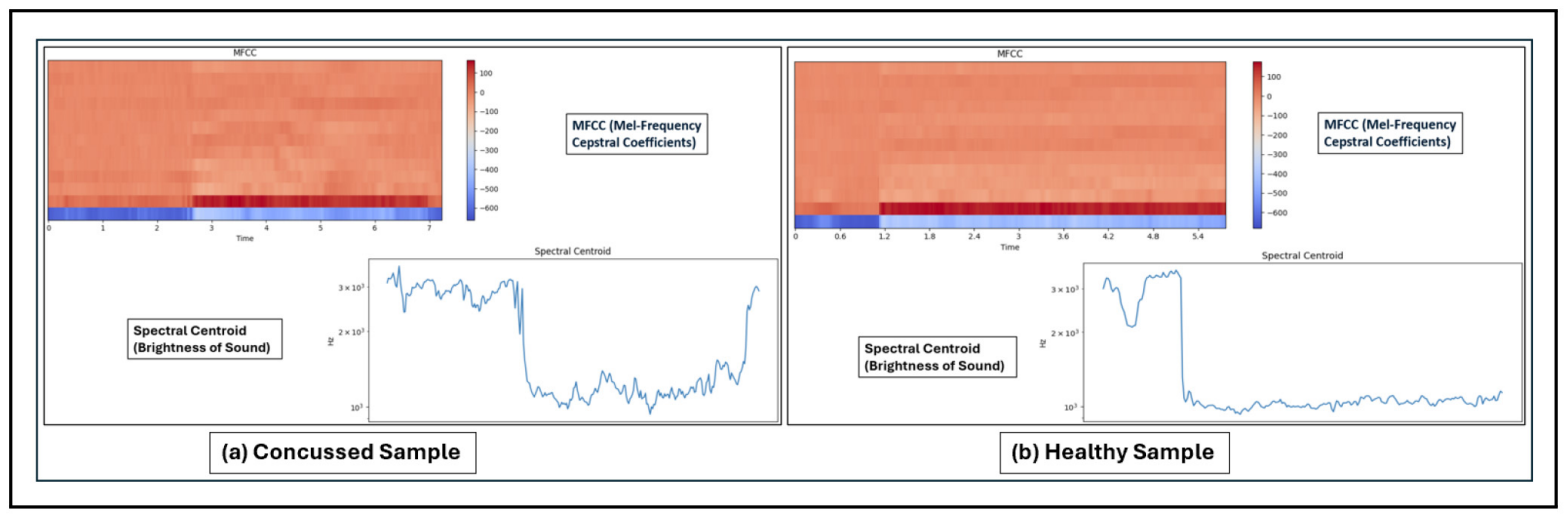
MFCC and spectral centroid visualizations for Group 3 sustained vowel tasks: **(a)** Concussed and **(b)** Healthy samples.

Figure 6 illustrates representative MFCC and spectral centroid patterns for healthy and concussed participants. Visual inspection suggests that sustained vowel segments are relatively stationary and lack the dynamic temporal variation present in articulation-rich tasks. Although some spectral instability is visible in concussed samples, these differences appear subtle.

Additional comparisons of spectral and temporal features between healthy and concussed recordings are summarized in Table 5. These observations suggest that sustained vowels provide weaker discriminative information for CNN-based classification compared to dynamic speech tasks.

**Table 5:** Comparison of selected spectral and temporal features between healthy and concussed participants in Group 3 tasks.

Feature	Baseline (healthy)	Concussed	Interpretation/implication
MFCC	Stable horizontal bands starting ~1.2 s; clear transitions from silence to vowel sound.	Similar bands starting later (~3 s) with slightly less sharp transitions.	Concussed vowels show less defined spectral characteristics.
Spectral centroid	Sharp drop from ~3,000 to ~1,000 Hz at vowel onset.	Similar drop but more fluctuating and irregular.	Indicates mild vocal instability or irregular articulation.
Spectral roll-off	Sharp onset drop and stable low-frequency roll-off.	Less sharp onset drop with more variability.	Suggests spectral variability related to vocal control.
Zero crossing rate	Rapid drop to stable low values indicating voiced vowel.	Less stable baseline with fluctuations.	Indicates irregularities in voicing and periodicity.
Spectral contrast	Distinct harmonic bands and stable contrast.	Less distinct and more diffuse spectral contrast.	Reflects reduced harmonic clarity.

### 3.5 Group-wise Grad-CAM analysis

To further interpret model behavior across different speech tasks, Grad-CAM analysis was performed to visualize the regions of mel spectrograms that contributed most strongly to classification decisions.

**Figure 7:**
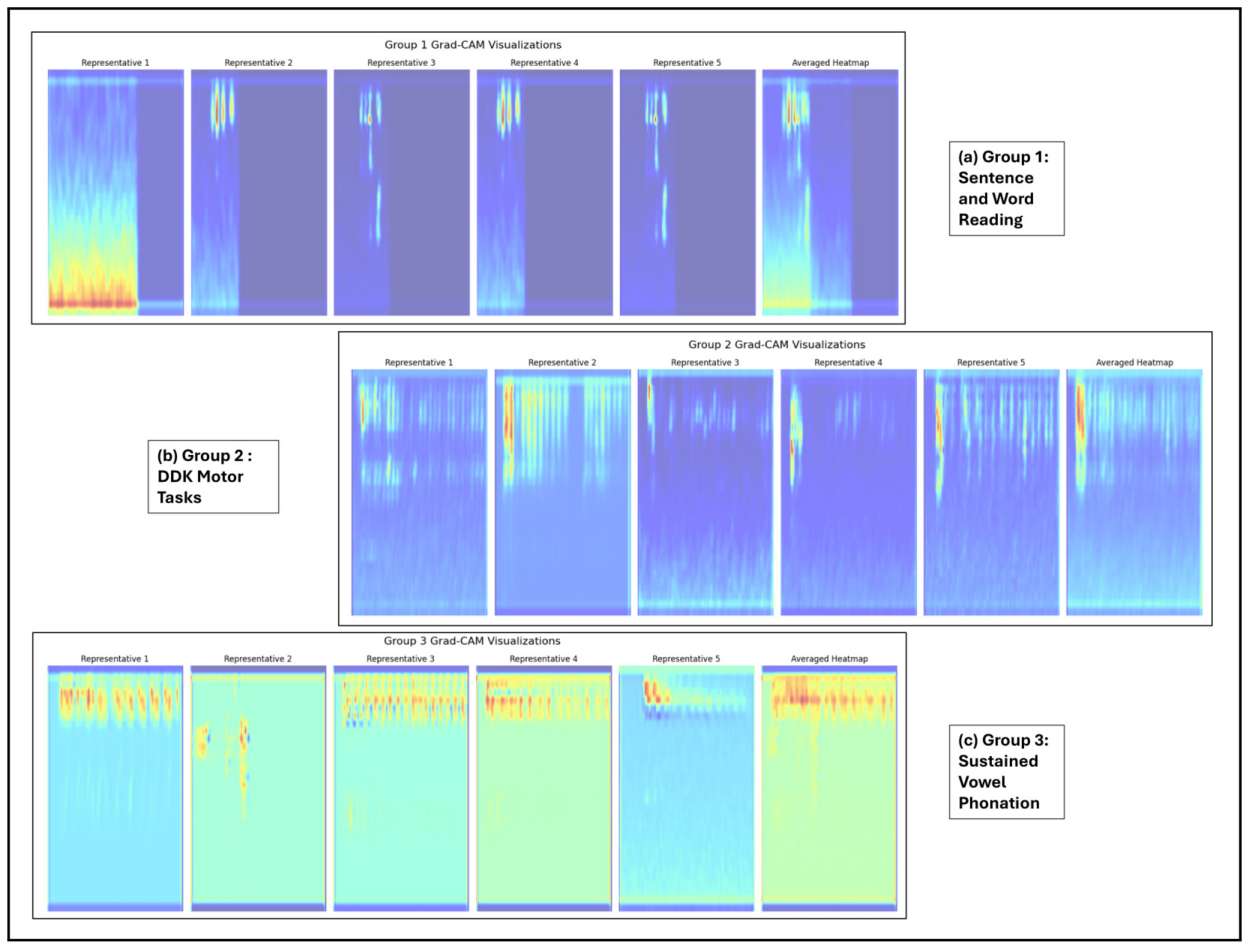
Grad-CAM visualizations across the three groups of neurological speech tests: **(a)** Group 1, **(b)** Group 2, and **(c)** Group 3.

Figure 7 shows representative Grad-CAM visualizations for each speech task group. For Group 1 (sentence and word reading tasks), the CNN consistently focused on early temporal regions and mid-to-high spectral bands corresponding to phonetic transitions and consonant bursts.

For Group 2 (DDK motor tasks), the model concentrated on rhythmic temporal patterns associated with rapid syllable repetitions. These attention patterns align with the motor coordination demands of the tasks.

In contrast, Grad-CAM visualizations for Group 3 (sustained vowel phonation) appeared more diffuse and inconsistent. The CNN showed scattered attention across the spectrogram without strong focus on vowel formant regions, reflecting the limited temporal dynamics of sustained vowel tasks. This observation aligns with the lower classification performance observed for Group 3 tasks (Table 6).

**Table 6:** Comparative analysis of recent studies for speech-based concussion detection.

References	Dataset	Data augmentation	Data type	Methodology	Accuracy	Shortcomings
Wall et al. (13)	40 athletes	None	Speech recordings	MFCC + BiLSTM	94.7% sensitivity	Small dataset
Tauro et al. (14)	1,210 samples	None	Short voice segments	DL on raw audio	85%	Focus on RHI
Thanjavur et al. (32)	58 athletes	None	EEG signals	LSTM on EEG	94%	Requires specialized equipment
Our study	225 participants	Gaussian noise	Speech recordings	Mel spectrogram + CNN	Avg 91.75%	Dataset expansion needed

## 4 Discussion

### 4.1 Key findings and interpretation

This study evaluated whether structured speech recordings could be used to distinguish athletes with clinically confirmed concussion from healthy control or baseline recordings using mel spectrogram representations and a convolutional neural network. Across the eight speech tasks, the model achieved its strongest performance on tasks involving dynamic articulation, sentence or word reading, and rapid motor-speech coordination, whereas sustained vowel phonation tasks showed comparatively weaker performance. These findings suggest that the discriminative information available for speech-based concussion detection is task-dependent and that articulation-rich speech tasks may provide more useful acoustic cues than relatively stationary phonatory tasks.

A clinically relevant interpretation of this pattern is that concussion-related speech changes may be more readily revealed during tasks that require rapid articulatory transitions, temporal sequencing, prosodic modulation, and coordinated motor planning. Reading tasks and diadochokinetic (DDK) repetitions impose greater demands on speech timing, articulatory precision, and motor coordination than sustained vowels, and these demands may make subtle post-concussion disruption more observable in the acoustic signal (10, 12, 26–28). In contrast, sustained vowel tasks primarily reflect vocal steadiness and respiratory-phonatory control within a relatively stationary signal, which may provide fewer discriminative time–frequency variations for CNN-based classification.

This interpretation is consistent with the group-wise findings observed in this study. Group 1 (sentence and word reading tasks) and Group 2 (DDK motor-speech tasks) showed stronger classification performance than Group 3 (sustained vowel tasks), supporting the view that dynamic tasks are better suited for identifying subtle concussion-related speech changes. Rather than indicating that sustained vowels are clinically uninformative, these results suggest that such tasks may be less compatible with the current spectrogram-based CNN framework and may require alternative modeling approaches or complementary acoustic descriptors.

The confusion matrix analysis further showed that performance differences across tasks were not solely a matter of overall accuracy, but also reflected different balances between false positives and false negatives. For example, some tasks produced highly conservative behavior with very high precision but somewhat lower recall, while others showed a more balanced trade-off. In a clinical context, such task-specific behavior may become important when speech-based models are eventually used as adjunct tools for screening rather than as stand-alone diagnostic systems.

Importantly, the present model should be interpreted as identifying speech changes associated with concussion within this dataset, rather than diagnosing structural brain injury, localizing pathology, or grading injury severity.

### 4.2 Interpretation of task-specific model behavior

The Grad-CAM visualizations provide additional qualitative insight into why certain tasks may have supported better classification performance. For Group 1 and Group 2 tasks, the CNN tended to focus on localized spectro-temporal regions associated with phonetic transitions, rhythmic articulatory bursts, and rapidly changing acoustic structure. These regions are consistent with the types of speech events that may reflect altered timing, sequencing, and coordination following concussion. Because concussion may affect the integration of cognitive and motor-speech processes, it is plausible that these dynamic portions of the signal contain more informative markers than acoustically steady segments.

By contrast, the Grad-CAM patterns for sustained vowel tasks were more diffuse and less consistently localized. This qualitative difference aligns with the lower performance observed for Group 3 and supports the interpretation that stationary phonation may provide weaker discriminative structure for the current model. However, these Grad-CAM findings should be interpreted cautiously. In the present study, Grad-CAM was used as a qualitative interpretability tool to generate insight into model attention patterns rather than as a quantitative validation of specific neurophysiological mechanisms. Accordingly, the interpretability results are best viewed as supportive and hypothesis-generating rather than definitive.

The exploratory feature comparisons for sustained vowels lead to a similar conclusion. Although subtle differences in spectral stability and temporal regularity were observed between concussion and control recordings, these differences were modest relative to the stronger temporal variation seen in articulation-rich tasks. Taken together, the qualitative feature analysis and Grad-CAM visualizations suggest that the lower performance of sustained vowels likely reflects both weaker task informativeness and limited compatibility with the present CNN-based modeling framework.

### 4.3 Comparison with prior studies and methodological contributions

This study contributes to the literature on speech-based concussion detection by applying a mel-spectrogram-based CNN to full-length recordings from eight structured neurological speech tasks. Prior work in this area has often relied on handcrafted acoustic features combined with traditional machine learning models, or on shorter speech samples and narrower task sets (11–14, 31). In contrast, the present work evaluates multiple task types within a unified deep learning framework and explicitly compares their relative discriminative value.

An important contribution of this study is therefore not only the classification performance itself, but also the task-level comparison. The results consistently indicate that articulation-rich reading tasks and DDK repetitions provide stronger discriminative information than sustained vowel tasks within this dataset. This task-sensitive perspective is clinically meaningful because it suggests that speech-based concussion assessment may benefit from carefully selecting or prioritizing speech tasks that place greater demands on timing, sequencing, and motor coordination.

At the same time, the findings should be interpreted in the context of the study design. Although the proposed CNN performed well within this dataset, the present work did not include a direct comparison against internal baseline models such as handcrafted-feature-based classifiers or simpler neural architectures. As a result, the study supports the promise of the proposed approach but does not establish that it is definitively superior to all alternative methods on this dataset. Future work should include explicit baseline comparisons to better contextualize the benefits of the mel-spectrogram-based CNN framework.

### 4.4 Limitations and future work

Several limitations should be considered when interpreting these findings. First, the study was conducted in a relatively specific population of high school and collegiate athletes, and the model was developed for concussion vs. control classification within this context. Accordingly, the results should be interpreted as a proof-of-concept investigation in a young athlete cohort rather than as evidence of broad applicability to neurological assessment in general. Extension to other populations, age groups, injury mechanisms, or clinical settings will require further validation.

Second, the current study did not include external validation on an independent dataset. Performance was estimated using participant-level cross-validation within the available cohort, which provides useful internal evidence but does not fully establish generalizability across institutions, devices, environments, or populations. External evaluation will be important in future work to determine how robust the learned patterns are beyond the present dataset.

Third, the final analytic sample size varied somewhat across speech tasks after quality filtering, and the overall dataset remains modest for deep learning applications. Although augmentation helped increase sample diversity, larger datasets with broader demographic and clinical diversity will be necessary to strengthen robustness and reduce uncertainty in performance estimates.

Fourth, the present study did not include direct baseline-model comparisons. While prior literature provides useful context, future work should compare the proposed CNN more directly with conventional machine learning methods based on handcrafted acoustic features, as well as with alternative deep learning architectures. Such comparisons would help clarify whether the observed gains are attributable to the mel-spectrogram representation, the CNN architecture, the task design, or their combination.

Fifth, the interpretability analysis was primarily qualitative. Grad-CAM offered useful insight into model attention patterns, but it does not by itself confirm the physiological source of the discriminative cues. Future work could strengthen interpretability by incorporating quantitative analyses of attention concentration, formal feature-attribution comparisons, or targeted acoustic analyses linked to established speech-motor measures.

Finally, although the recordings were collected under relatively controlled acquisition conditions, real-world deployment would require robustness to background noise, device variability, spontaneous speech, and less standardized testing settings. Future work should therefore examine how task performance changes under more naturalistic acquisition conditions and whether shorter or more practical task batteries can retain sufficient discriminatory value for screening applications.

### 4.5 Conclusions and clinical implications

Within the present dataset, the results demonstrate that structured speech tasks can provide useful acoustic information for distinguishing concussion from control recordings using a CNN-based mel-spectrogram framework. The strongest performance was obtained from tasks involving dynamic articulation and motor-speech coordination, whereas sustained vowel tasks were less informative in this setting. From a practical standpoint, these task-specific findings suggest that future speech-based concussion screening tools may benefit from prioritizing dynamic articulation and motor-speech tasks, while using sustained vowels as complementary rather than primary inputs. These findings reinforce the importance of task selection in speech-based concussion assessment and suggest that articulation-rich tasks may be more suitable than stationary phonation tasks for this type of modeling.

At the same time, the present findings should be interpreted conservatively. This study does not establish a general neurological screening tool, nor does it replace clinical evaluation. Rather, it provides proof-of-concept evidence that structured speech analysis may serve as a useful non-invasive complement to concussion assessment in a defined athlete population. With larger and more diverse datasets, external validation, direct baseline comparisons, and more quantitative interpretability analyses, speech-based AI systems may become increasingly valuable as supportive tools for concussion screening and monitoring.

## Data Availability

The data analyzed in this study is subject to the following licenses/restrictions: Due to privacy and IRB restrictions, these data cannot be shared publicly. Researchers interested in access may need to obtain appropriate approvals from the original data custodians. Requests to access these datasets should be directed to cpoellab@fiu.edu.
